# Use of health-related quality of life measures to predict health utility in postmenopausal osteoporotic women: results from the Multiple Outcomes of Raloxifene Evaluation study

**DOI:** 10.1186/1477-7525-11-189

**Published:** 2013-11-05

**Authors:** Russel Burge, Wei Shen, April N Naegeli, Jahangir Alam, Stuart Silverman, Deborah T Gold, Tsehua Shih

**Affiliations:** 1 Eli Lilly and Company, Indianapolis, IN, USA; 2 Cedars Sinai Medical Center, Beverly Hills, CA, USA; 3 Duke University Medical Center, Durham, NC, USA; 4 inVentiv Clinical Solutions, Hunt Valley, MD, USA

**Keywords:** Health utility, Postmenopausal osteoporosis, HRQoL, OPAQ

## Abstract

**Background:**

The aim of this study is to examine the associations between health utility (HU), health-related quality of life (HRQoL), and patient characteristics in postmenopausal osteoporotic (PMO) women.

**Methods:**

Baseline data from a subsample of 1,245 participants of the Multiple Outcomes of Raloxifene Evaluation study, a randomized, placebo-controlled, multinational clinical trial to evaluate the safety and efficacy of raloxifene, were analyzed. The study cohort included 694 participants from non-European Union (non-EU) countries and 551 participants from EU countries. All participants with complete baseline HU and HRQoL assessments were included in the following analyses: 1) HU (HUI or EQ-5D) and HRQoL (QualEFFO or OPAQ and NHP) associations; 2) HU variability explained by HRQoL domains; and 3) the percentage of HU variability explained by statistically significant (p < 0.05) HRQoL domains, after adjusting for baseline characteristics.

**Results:**

Several domains were significantly associated with HU scores. HU variance was well explained (41% to 61%) by 4 to 6 (p < 0.05) significant HRQoL domains. After controlling for baseline characteristics, 48% to 64% of the HU variance was well explained by 5 to 7 significant (p < 0.05) HRQoL domains. Additional trend analyses detected statistically significant decreases in HRQoL and HU scores with an increased number of vertebral and non-vertebral fractures.

**Conclusions:**

Both disease-targeted and generic HRQoL domains were well correlated with HU. A large percentage (48% to 64%) of the HU variance was explained by HRQoL, after adjusting for baseline characteristics. Both disease-targeted and generic HRQoL measures were significant predictors of HU. HRQoL and HU scores decreased with increased vertebral and non-vertebral fractures.

## Background

Osteoporosis is characterized by low bone mass and micro-architectural deterioration of bone tissue, which leads to increased bone fragility and risk of fracture. Osteoporosis is a major public health threat, affecting an estimated 200 million postmenopausal osteoporotic (PMO) women worldwide [1,2]. Osteoporosis influences multiple dimensions of HRQoL [3-7]. Vertebral fractures in PMO women can result in acute and chronic back pain, declines in physical functioning, and psychosocial impairments [8-14].

Previous research has reported that lumbar vertebral fractures, in particular, are associated with a reduced HRQoL, as measured by the Osteoporosis Assessment Questionnaire (OPAQ) [9,15,16]; the European Union (EU) Foundation of Osteoporosis Quality-of-Life Assessment (QualEFFO) [7,16]; and, to a lesser degree, the Nottingham Health Profile (NHP) and EQ-5D [11].

Fractures related to osteoporosis may also have an impact on HU scores. HUs are measured on a scale of 0 to 1, with 0 being death and 1 being the best state of health (values can drop below 0, which represents a state worse than death; however, values cannot rise above 1) [17]. HU values provide preference-based measures of utility that are used to derive quality-adjusted life years (QALYs), which are standardized outcome measures applicable to any therapeutic area, used in cost-utility analyses (incremental costs divided by incremental QALYs) in economic evaluations of competing healthcare interventions [18].

Marciniak et al. reported that in a group of women initiating and continuing treatment with bone loss medications, significantly lower EQ-5D (HU) scores were associated with prior vertebral fractures, a fear of falling, a number of ongoing comorbid conditions, a higher body mass index (BMI), ongoing depression, and initiating or switching treatments at baseline [19].

Although general measures of HRQoL have been widely used in studies of osteoporosis, much less is known about the relationship between disease-targeted HRQoL instruments and preference-based HU instruments.

Recent research published by McDonough et al. presented models that were developed to predict HU (from estimates of EQ-5D [US version]) and the SF-6D, which is a 6-dimensional health state classification model created using the Medical Outcomes Study Short Form (36) Health Survey (SF-36) [20]. Health state utility values (HSUVs/scores) for either EQ-5D or SF-6D were generated using OPAQ data in the models created [20]. This research provided a first step in predicting HU scores (in this case EQ-5D and the SF-6D HSUV estimates) using HRQoL scores (in this research, the OPAQ disease-targeted measure) when only HRQoL clinical trial data are available [20].

The purpose of our study was to examine associations between HRQoL, HU, and patient characteristics in PMO women. In addition, we were interested in determining the potential for 2 disease-targeted, and 1 generic HRQoL/health status measure to predict HU in PMO women with vertebral and non-vertebral fractures. We hypothesized that the disease-targeted, generic, and health status measures would correlate with 2 different HU measures in PMO women, given the overlap in some items within these instruments. Disease-targeted instruments may study novel domains which may not be reflected in a generic instrument, and could result in the generic instrument being less sensitive to a specific disease event and subsequent outcomes. Therefore, this research may provide new evidence to support this hypothesis, as well as new information to help better characterize the associations between these classes of instruments. The study also may provide new insights on the underlying domain constructs for osteoporosis HRQoL instruments.

## Methods

### Data source

A subsample of 1,245 PMO women participating in the Multiple Outcomes of Raloxifene Evaluation (MORE) study, which was a randomized, placebo-controlled, multinational clinical trial to evaluate the safety and efficacy of raloxifene [21], was utilized as the data source.

Participants in MORE included 7,705 women who had been PMO for at least 2 years and who were up to 80 years of age (mean age 66.5 years) with osteoporosis, defined as low bone mineral density (BMD) or radiographically apparent vertebral fractures [21]. The first patient visit in the MORE study was in December 1994, while the last patient visit occurred in September 1998. The MORE study protocol was approved by the human studies review board at each center, and informed consent was obtained [21].

### Study population

All participants with complete baseline HRQoL and HU assessments were included in the analyses (all HRQoL and HU assessments were administered at study baseline and at months 12, 24, and 36). Of the original 7,705 MORE study subjects, 694 participants were from the non-European Union (non-EU) countries of Australia (n = 53), Canada (n = 110), New Zealand (n = 16), and the United States (n = 515), and 551 subjects were from the EU countries of Belgium (n = 38), United Kingdom (n = 141), the Netherlands (n = 319), and Sweden (n = 53).

### Major measurements

#### HRQoL measurements

For participants in the EU cohort, disease-targeted HRQoL was assessed with the QualEFFO, a 54-item instrument, which assesses 6 domains of HRQoL, including pain, daily activity, mobility, general health, mental health, and overall quality of life [8,11]. The instrument is disease-targeted with response options on 5-point Likert scales and visual analog scales (VASs). The QualEFFO scores range from 0 to 100, with higher scores indicating better HRQoL (higher scores: 100 = better for the original 54-item QualEFFO instrument used in this study) [8,11]. The additional items, which are not on the current QualEFFO instruments, were derived from questionnaires used in the Mediterranean Osteoporosis Study and the European Vertebral Osteoporosis Study [22]. The instrument has been shown to have good test-retest reliability (kappa 0.54 to 0.90) and internal consistency (Crohnbach’s α 0.72 to 0.92) and demonstrates the ability to discriminate between women with vertebral fractures and without fracture [23].

For subjects in the non-EU cohort, disease-targeted HRQoL was assessed with the OPAQ, a 67-item instrument that assesses HRQoL in 4 dimensions (physical function, emotional status, symptoms, and social interactions) that cover the following 14 domains: walking/bending, sitting/standing, dressing/reaching, household/ self-care, transfers, usual work, fear of falls, level of tension, body image, independence, back pain, fatigue, social activity, and support, family and friends. Response options are based on a 4- to 5-point Likert scale, with a variety of response options including all days, most days, some days, few days, and no days [7,15,16]. The OPAQ scores range from 0 to 100, with higher scores indicating better HRQoL.

Generic HRQoL was assessed using the NHP for all EU and non-EU participants. The NHP is a 38-item instrument that provides a patient’s perception of HRQoL/health status [24]. Response options on the NHP provide dichotomous (Yes/No) indicators of emotional, social, and physical health problems experienced by participants. There are 6 NHP domains, including emotional reaction, energy, physical mobility, pain, sleep, and social interaction [24]. Generic HRQoL scores range from 0 to 100, with lower scores indicating lower levels of distress [24].

#### HU measurements

For the non-EU cohort, HU was assessed using the Health Utilities Index Mark 2 (HUI2), which is a preference-based generic measure of utility that measures health status. The HUI assesses a patient’s health in terms of ability or disability over the past 4 weeks [24]. There are 11 domains in the HUI, including reading, hearing, speaking, emotions/happiness, pain/discomfort, walking, use of hands/fingers, memory, cognition/problem solving, basic care, and worry [25]. Scores on the HUI can range from 0 (value for being dead) to 1 (value for perfect health), with higher HUI scores indicating better states of health.

For subjects in the EU cohort, HU was assessed using the EQ-5D, which covers 5 dimensions of health [23]. Respondents rate their current health state in the following 5 areas: mobility, self-care, usual activities, pain/discomfort, and anxiety/depression. In addition, participants compare their current health state to their health state last year at this time and rate as better, same, or worse, and they also provide VAS response options ranging from 0 (worst imaginable health state) to 100 (best imaginable health state).

### Statistical analyses

Subjects’ baseline demographic and clinical characteristics were summarized for both cohorts. Mean and standard deviation (SD) were calculated for continuous variables and proportions were reported for categorical variables. T-tests on continuous variables and Fisher exact tests on dichotomous variables were conducted to test the baseline characteristics between the EU and non-EU cohorts.

Correlations between HU scores and HRQoL domain scores were then conducted by using Pearson’s correlation. For the non-EU cohort, this correlation included assessment of OPAQ versus HUI and NHP versus HUI. For the EU cohort, this included assessment of QualEFFO versus EQ-5D and NHP versus EQ-5D. In order to derive the best independent determinants of HU and to identify any redundant HRQoL domains within a questionnaire, multiple stepwise regressions were conducted using HU as a dependent variable and HRQoL domain scores as independent variables. These analyses, which examined the percentage of HU variance explained by HRQoL domains, were conducted separately for the disease-targeted and generic HRQoL measures. Multiple regressions, which used HU as a dependent variable and baseline characteristics as independent variables, were conducted to identify significant baseline characteristics (Table 1). Multiple stepwise regressions, which used HU as a dependent variable and significant HRQoL predictors (domains with p < 0.05) from the screening step *plus* baseline characteristics (listed in Table 1) as the independent variables, were then conducted. The significance levels for variables to enter and exit the models were 0.05 and 0.10, respectively. The ANOVA and trend tests were conducted to compare HU scores and HRQoL domains and scores among subgroups with 0, 1, 2, and 3 or more fractures.

**Table 1:** Association between baseline subject characteristics and HU: EU and Non-EU cohorts

**Non-EU cohort**		**EU cohort**	
**Baseline characteristic**	**C**	**Baseline characteristic**	**C**
Age (years)	0.16	Age	0.34
Alcohol use (Yes)	−0.19	Alcohol use (Yes)	−1.03
Height (cm)	0.158*	Height (cm)	0.247
Weight (kg)	−0.115*	Weight (kg)	−0.385**
Country AU vs. US	−4.359*	Country BE vs. UK	−9.088*
Country CA vs. US	−0.570	Country NL vs. UK	−2.997
Country NZ vs. US	−1.056	Country SE vs. UK	−3.684
Smoke (Yes)	−5.204*	Smoke (Yes)	−8.712**
Prevalent vertebral fractures	−0.638	Prevalent vertebral fractures	−2.245**
Baseline femoral neck BMD (# of SDs < mean for females)	−0.885	Baseline femoral neck BMD (# of SDs < mean for females)	2.192
Number of preexisting conditions	−0.447***	Number of preexisting conditions	−1.434***
Number of ONF	−0.549	Number of ONF	0.751
Years postmenopausal	−0.095	Years postmenopausal	−0.305
Family history of osteoporosis (Yes)	−1.004	Family history of osteoporosis (Yes)	4.332
History of hysterectomy (Yes)	−0.008	History of hysterectomy (Yes)	−4.723*
Married (Yes)	−0.118	Married (Yes)	0.587
Years of education	0.347*	Years of education	0.370
Baseline LS BMD (# of SDs < mean for females)	0.472	Baseline LS BMD (# of SDs < mean for females)	−0.209
Variance explained	0.14	Variance explained	0.20

*Abbreviations:AU* Australia, *BMD* Bone mineral density (score), *BE* Belgium, *C* Regression coefficient, *CA* Canada, *EU* European Union, *LS* Lumbar spine, *NL* Netherlands, *Non-EU* Non-European Union, *NZ* New Zealand, *ONF* Osteoporotic non-vertebral fractures, *SD* Standard deviation, *SE* Sweden, *UK* Great Britain, *US* United States.

*p < 0.05, **p < 0.001, ***p < 0.0001.

## Results

### Patient characteristics

The mean age for the non-EU cohort was 68.0 years, and mean height, weight and BMI were 159.7 cm, 65.4 kg, and 25.7 kg/cm^2^, respectively (Table 2). For the EU cohort, the mean values for age height, weight and BMI were 67.5 years, 160.1 cm, 64.7 kg, and 25.2 kg/cm^2^, respectively. BMI was statistically, significantly higher in the non-EU cohort versus the EU cohort. Other statistically significant differences included a higher percentage of smokers and alcohol users, more years of education, and more with self-reported, preexisting medical conditions among the non-EU cohort compared to the EU cohort. The non-EU cohort reported a higher percentage of participants with 1 or more osteoporotic non-vertebral fractures and a higher baseline lumbar BMD score, but a lower percentage of femoral neck BMD t-scores (Table 2).

**Table 2:** Baseline subject demographic and clinical characteristics

**Baseline characteristic**	**Non-EU (n = 694)**	**EU (n = 551)**	**p-value**
	Mean (SD)	Mean (SD)	
Age (years)	68.0 (6.9)	67.5 (6.2)	0.1994
Height (cm)	159.7 (6.3)	160.1 (6.6)	0.3001
Weight (kg)	65.4 (11.0)	64.7 (9.9)	0.2467
Body mass index (kg/cm^2^)	25.7 (4.2)	25.2 (3.6)	0.0370*
Caucasian (n, %)	672 (96.8)	543 (98.5)	0.0619
Smoker (Yes) (n, %)	617 (88.9)	421 (76.4)	<0.0001**
Use of alcohol (Yes) (n, %)	556 (80.1)	406 (73.7)	0.0038*
Number of prevalent vertebral fractures (≥1) (n, %)	433 (62.4)	279 (50.6)	<0.0001**
Baseline femoral neck BMD (# of SDs < mean for females)	−2.3 (.6)	−2.2 (.6)	0.0022*
Number of osteoporotic non-vertebral fractures (≥1) (n, %)	366 (52.7)	202 (36.7)	<0.0001**
Family history of osteoporosis (Yes) (n, %)	212 (30.5)	157 (28.5)	0.0001**
History of hysterectomy (Yes) (n, %)	186 (26.8)	134 (24.3)	0.3281
Married (Yes) (n, %)	390 (56.2)	314 (57.0)	0.7732
Number of years postmenopause	20.3 (8.7)	19.4 (7.7)	0.0551
Baseline lumbar spine BMD (# of SDs < mean for females)	−2.4 (1.2)	−2.7 (1.1)	0.0001**
Number of years of education	13.6 (3.1)	10.1 (3.2)	<0.0001**
Number of self-reported preexisting conditions	10.9 (5.9)	5.9 (4.3)	<0.0001**

*Abbreviations*: *BMD* Bone mineral density, *EU* European Union, *Non-EU* Non-European Union, *SD* Standard deviation.

*p < 0.05, **p < 0.0001.

Table 3 provides baseline OPAQ, NHP and HUI mean scores for the non-EU cohort, and QualEFFO, NHP, and EQ-5D scores for the EU cohort. The mean utility scores were 0.85 for the non-EU cohort (based on the HUI) and 0.78 for the EU cohort (based on the EQ-5D).

**Table 3:** Summary of baseline EQ-5D, QualEFFO, OPAQ, HUI, and NHP domains/scores: EU cohort and Non-EU cohorts

**Instrument (Cohort) domain**	**N**	**Mean**	**SD**	**Min**	**Max**
**OPAQ (Non-EU)**					
Walking/Bending	693	85.42	17.08	7.14	100.00
Standing/Sitting	692	80.16	20.83	0.00	100.00
Dressing/Reaching	691	93.10	14.76	0.00	100.00
Household/Self Care	692	93.16	12.60	31.25	100.00
Transfers	693	90.66	18.32	0.00	100.00
Usual Work	692	92.02	15.87	0.00	100.00
Fear of Falls	692	73.47	19.44	10.00	100.00
Level of Tension	690	67.93	17.38	15.00	100.00
Body Image	693	67.26	24.77	0.00	100.00
Independence	691	83.83	17.19	0.00	100.00
Back Pain	694	73.71	23.69	0.00	100.00
Fatigue	693	62.52	18.75	0.00	100.00
Social Activity	692	40.44	19.40	0.00	100.00
Support, Family and Friends	691	85.49	20.26	0.00	100.00
**NHP (Non-EU)**					
Emotional Reaction	681	5.08	12.57	0.00	100.00
Energy	688	11.55	23.33	0.00	100.00
Physical Mobility	686	9.54	13.42	0.00	67.16
Pain	678	11.88	21.37	0.00	100.00
Sleep	687	19.47	25.06	0.00	100.00
Social Interaction	684	2.73	9.87	0.00	79.87
**HUI (Non-EU) Utility Score**	694	0.85	0.12	0.22	1.00
**QualEFFO (EU)**					
Pain	541	68.77	27.20	0.00	100.00
Daily Activity	550	91.20	13.83	11.11	100.00
Mobility	550	77.90	19.61	0.00	100.00
General Health	548	58.25	20.92	0.00	100.00
Mental Health	549	74.95	16.84	12.50	100.00
Overall QOL	524	73.42	20.13	2.00	100.00
**NHP (EU)**					
Emotional Reaction	525	10.07	18.58	0.00	100.00
Energy	547	16.64	29.62	0.00	100.00
Physical Mobility	533	14.02	18.47	0.00	100.00
Pain	526	18.93	26.42	0.00	100.00
Sleep	536	24.37	28.63	0.00	100.00
Social Interaction	533	5.38	14.88	0.00	100.00
**EQ-5D (EU) Utility Score**	551	0.78	0.23	−0.59	1.00

*Abbreviations*: *EU* European Union, *HUI* Health Utility Index, *Max* Maximum score, *Min* Minimum score, *NHP* Nottingham Health Profile, *Non-EU* Non-European Union, *OPAQ* Osteoporosis Assessment Questionnaire, *QOL* Quality of life, *QualEFFO* EU Foundation of Osteoporosis Quality-of-Life Assessment, *SD* Standard deviation.

Scoring: EQ-5D scores: 0 (worst) - 1.0 (best); HUI scores: 0 (worst) - 1.0 (best); NHP scores: 0 (best) - 100 (worst); OPAQ scores: 0 (worst) - 100 (best), QualEFFO scores (54-item QualEFFO): 0 (worst) - 100 (best).

### Correlations between HRQoL and HU measures

The HRQoL domains were well correlated with HU scores, especially for domains related to pain, physical function, and emotional and mental health, which are core domains of the EQ-5D and HUI (Table 4). For OPAQ, 8 domains had correlations with HUI ≥ 0.40:

•4 from the physical function dimension: walking/bending, standing/sitting, transfers, and usual work;

•2 from the emotional status dimension: fear of falls and independence; and

•2 from the symptoms dimension: back pain and fatigue.

**Table 4:** Correlations between HRQoL and HU measures: EU and Non-EU cohorts

**Non-EU cohort**				**EU cohort**			
**OPAQ vs. HUI**		**NHP vs. HUI**		**QualEFFO vs. EQ-5D**		**NHP vs. EQ-5D**	
**OPAQ domain**	**r**	**NHP Domain**	**r**	**QualEFFO domain**	**r**	**NHP domain**	**r**
Walking/Bending	0.524	Emotional reaction	−0.393	Pain	0.571	Emotional reaction	−0.561
Standing/Sitting	0.490	Energy	−0.477	Daily activity	0.713	Energy	−0.593
Dressing/Reaching	0.325	Physical mobility	−0.506	Mobility	0.657	Physical mobility	−0.596
Household/Self care	0.335	Pain	−0.578	General health	0.558	Pain	−0.650
Transfers	0.416	Sleep	−0.386	Mental health	0.540	Sleep	−0.335
Usual work	0.400	Social interaction	−0.272	Overall QOL	0.515	Social interaction	−0.347
Fear of falls	0.456						
Level of tension	0.342						
Body image	0.265						
Independence	0.405						
Back pain	0.495						
Fatigue	0.444						
Social activity	0.116*						
Support, family and friends	0.093*						

*Abbreviations:EU* European Union, *HUI* Health Utility Index, *NHP* Nottingham Health Profile, *Non-EU* Non-European Union, *OPAQ* Osteoporosis Assessment Questionnaire, *QOL* quality of life, *QualEFFO* EU Foundation of Osteoporosis Quality-of-Life Assessment, *r* = Pearson’s correlation coefficient estimate.

Scoring: OPAQ scores: 0 (worst) - 100 (best), HUI scores: 0 (worst) - 1.0 (best), NHP scores: 0 (best) - 100 (worst), EQ-5D scores: 0 (worst) - 1.0 (best). QualEFFO scores (54-item QualEFFO): 0 (worst) - 100 (best).

*p < 0.05, p < 0.0001 for all other coefficients.

The OPAQ domains of social activity and support, family and friends, both from the social interaction dimension, had the weakest correlations with HUI (Table 4). All correlations between NHP and HUI were negative, with higher utility values indicating less distress.

All QualEFFO domains had strong correlations (>0.50) with EQ-5D utility scores (Table 4). For NHP, all correlations with EQ-5D were negative, with higher HU indicating less distress.

In both the non-EU and EU cohorts, pain, physical mobility, and energy had correlations >0.40 with HU (Table 4). In addition, the EU cohort had a correlation >0.40 in the emotional reaction domain.

The associations between disease-targeted HRQoL and HU (OPAQ vs. HUI and QualEFFO vs. HUI), from a clinical point of view, were similar to the associations between generic HRQoL and HU (NHP vs. HUI and NHP vs. EQ-5D) in both cohorts (Table 4).

### Association between baseline patient characteristics and HU

In the non-EU cohort, BMI, country = Australia (vs. United States), smoke, and number of preexisting conditions were negatively associated with HU, while years of education were positively associated with HU (Table 1). In the EU cohort, BMI, country = Belgium (vs. United Kingdom), smoke, prevalent vertebral fractures, number of preexisting conditions, and history of hysterectomy were negatively associated with HU.

In the analyses of association between baseline patient characteristics and HU, the variability in HU did not appear to be explained by baseline characteristics, as HU variability explained by baseline characteristics was only 13% for non-EU and 19% for EU cohorts, respectively (Table 1).

### Stepwise regression to identify independent HRQoL predictors of HU

In the multiple stepwise regression models, prior to adjusting for baseline characteristics, where associations between HU and HRQoL were examined, multiple HRQoL domains were found to be significantly associated with HU. A large percentage of the variance in HU was explained by HRQoL domains (Table 5). For the non-EU cohorts, 5 OPAQ domains (walking/bending, back pain, level of tension, fear of falls, and fatigue) were significantly associated with HU, and together they explained 41% of the variance in HU. All 6 NHP domains (pain, emotional reaction, physical mobility, sleep, energy, and social interaction) were significantly associated with HU, and together they explained 44% of the variance in HU. For the EU cohort, 4 QualEFFO domains (daily activity, mental health, pain, and mobility) were significantly associated with EQ-5D utility scores, and together they explained 61% of the variance in HU. Four NHP domains (pain, emotional reaction, physical mobility, and energy) were significantly associated with EQ-5D utility scores, and together they explained 53% of the variance in HU.

**Table 5:** Significant HRQoL predictors of HU from stepwise regression model without adjustment for baseline patient characteristics: EU and Non-EU cohorts

**Non-EU Cohort**				**EU Cohort**			
**OPAQ vs. HUI**		**NHP vs. HUI**		**QualEFFO vs. EQ-5D**		**NHP vs. EQ-5D**	
**n = 677**		**n = 662**		**n = 538**		**n = 495**	
***OPAQ domain***	**C**	***NHP domain***	**C**	***QualEFFO domain***	**C**	***NHP Domain***	**C**
Walking/Bending	0.184	Pain	−0.188	Daily activity	0.689	Pain	−0.275
Back pain	0.097	Emotional reaction	−0.104	Mental health	0.327	Emotional reaction	−0.326
Level of tension	0.084	Physical mobility	−0.139	Pain	0.158	Physical mobility	−0.274
Fear of falls	0.101	Sleep	−0.067	Mobility	0.132*	Energy	−0.089*
Fatigue	0.077*	Energy	−0.055*				
		Social interaction	−0.099*				
Variance explained	0.41	Variance explained	0.44	Variance explained	0.61	Variance Explained	0.53

*Abbreviations*: *C* Regression coefficient, *EU* European Union, *HUI* Health Utility Index, *NHP* Nottingham Health Profile, *Non-EU* Non-European Union, *OPAQ* Osteoporosis Assessment Questionnaire, *QualEFFO* EU Foundation of Osteoporosis Quality-of-Life Assessment.

Scoring: OPAQ scores: 0 (worst) - 100 (best), HUI scores: 0 (worst) -1.0 (best), NHP scores: 0 (best) - 100 (worst), EQ-5D scores: 0 (worst) - 1.0 (best), QualEFFO scores (54-item QualEFFO): 0 (worst) - 100 (best).

*p < 0.05, p < 0.0001 for all other coefficients.

### Determinants of HU, with adjustment for baseline patient characteristics

In the final multiple regression model, where HU was the dependent variable and significant HRQoL domains from the screening step *plus* baseline patient characteristics were included as independent variables, multiple disease-targeted and generic HRQoL domains were found to be significantly associated with HU (Table 6). For the non-EU cohort, 3 OPAQ domains (walking/bending, fear of falls, and fatigue) and 4 NHP domains (emotional reaction, pain, sleep, and social interaction) were significantly associated with HU, and together this model explained 48% of the variance in HU. For the EU cohort, 3 QualEFFO domains (pain, daily activity, and mental health) and 2 NHP domains (emotional reaction and pain) were significantly associated with HU, and together this explained 64% of the variance in HU.

**Table 6:** Significant HRQoL predictors of HU from stepwise regression model with adjustment for baseline patient characteristics

**Non-EU cohort**		**EU cohort**	
**Dependent Var = HUI; Independent Vars = OPAQ, NHP, BD**		**Dependent Var = EQ-5D; Independent Vars = QualEFFO, NHP, BD**	
**Domain**	**C**	**Domain**	**C**
OPAQ Walking/Bending	0.137***	QualEFFO pain	0.108**
OPAQ fear of falls	0.071**	QualEFFO daily activity	0.644***
OPAQ fatigue	0.057*	QualEFFO mental health	0.141*
NHP emotional reaction	−0.096***	NHP emotional reaction	−0.299***
NHP pain	−0.169***	NHP pain	−0.154***
NHP sleep	−0.056***		
NHP social interaction	−0.109*		
Variance explained	0.48	Variance explained	0.64

*Abbreviations*: *BD* Baseline demographic (characteristic), *C* Regression coefficient *EU*, European Union, *HUI* Health Utility Index, *NHP* Nottingham Health Profile, *Non-EU* Non-European Union, *OPAQ* Osteoporosis Assessment Questionnaire, *QualEFFO* EU Foundation of Osteoporosis Quality-of-Life Assessment, *Var* variable.

Scoring: OPAQ scores: 0 (worst) - 100 (best), HUI scores: 0 (worst) - 1.0 (best), NHP scores: 0 (best) - 100 (worst), EQ-5D scores: 0 (worst) - 1.0 (best), QualEFFO scores (54-item QualEFFO): 0 (worst) - 100 (best).

*p < 0.05, **p < 0.001, ***p < 0.0001.

Additional analyses in both cohorts examined the associations between the number of prevalent fractures (ie, vertebral and non-vertebral) and disease-targeted HRQoL scores, domain scores, and HU scores. (Please see Additional file 1 and Additional file 2 for results.) These analyses suggested a decrease in both HRQoL and HU scores, with an increase in the number of vertebral and non-vertebral fractures. There was a statistically significant, direct linear relationship between the number of vertebral fractures (0, 1, 2, 3+) and a reduction in HRQoL scores for those in the non-EU and EU cohorts, based on OPAQ, QualEFFO, NHP, and EQ-5D scores. The OPAQ walking/bending domain scores in the non-EU cohort, for example, were 87.4, 86.4, 84.8, and 76.8 for 0, 1, 2, and 3+ vertebral fractures (p < 0.001), while HUI scores in subjects with 0 or 1 vertebral fracture declined from 0.9 to 0.8 with 2 or 3+ vertebral fractures (p = 0.006) (see Additional file 1). There were also statistically significant, linear relationships between the non-vertebral fractures (0, 1, 2, 3+) and reductions in HRQoL and HU for those in the non-EU and EU cohorts, although the number of significant domains and strength of statistical significance was less pronounced for those with non-vertebral fractures than it was for those with vertebral fractures (see Additional file 2).

## Discussion

Baseline data from a subsample of 1,245 women, who participated in the MORE study, was utilized to examine the association between HU and HRQoL, as well as patient characteristics.

Results of this study revealed that there were multiple HRQoL domains that were significantly correlated with HU scores. More specifically, disease-targeted and generic HRQoL domains were found to be well correlated with HU scores, especially for domains related to pain, physical function, and emotional and mental health, which are basic domains of the EQ-5D and HUI utility scores.

In the multiple stepwise regression models, where associations between HU and HRQoL were examined, multiple HRQoL domains were found to be significantly associated with HU. A large percentage (41% to 61%) of the variance of HU scores was explained by HRQoL domains. After adjusting for baseline patient characteristics, the proportion of variance in HU scores explained by HRQoL domains improved slightly to 48% to 64%, which indicates that baseline patient characteristics were not major confounding factors, and that both disease-targeted and generic HRQoL measures were significantly correlated with HU.

In the final multiple regression models, with HU as the dependent variable and the significant HRQoL domains from the screening step *plus* the significant baseline patient characteristics included as independent variables, there were multiple HRQoL domains significantly associated with HU. These significant domains, from both the disease-targeted and generic HRQoL measures, complement each other and encompass the major concepts covered in both HU measures, including pain, physical function, and emotional and mental health. After adjusting for baseline patient characteristics, both disease-targeted and generic HRQoL measures remain significantly correlated with utility scores.

In addition, the number of prevalent fractures was significantly correlated with both HRQoL and HU scores. Prevalent fractures, particularly the number of vertebral fractures, appeared to significantly impact both HRQoL (OPAQ and QualEFFO) scores, as well as HU (HUI and EQ-5D) scores. As the number of vertebral fractures increased, HRQoL and HU scores decreased, as measured by the OPAQ, QualEFFO, HUI, and EQ-5D.

In general, findings were similar between the 2 cohorts, despite the different utility and disease-targeted HRQoL measures. The associations between HRQoL and HU appeared to be stronger in the EU population (as measured by the QualEFFO and EQ-5D), compared to the non-EU population. This result could be due to underlying unmeasured differences between the 2 populations or to the different HU and HRQoL instruments used.

While it is difficult to compare the findings between the non-EU and EU cohorts directly, given that 2 different utility measures were used (HUI and EQ-5D), other researchers have shown the correlation between EQ-5D and HUI to be high, 0.71 and 0.67, respectively [26,27]. Characterizing the associations between the OPAQ and HUI; the QualEFFO and EQ-5D; and the OPAQ, QualEFFO, and NHP have been original contributions to the field.

Our results for the non-EU cohort were similar to those reported by Marciniak et al. [19]. In particular, the EQ-5D HU scores were similar between studies, as Marciniak et al. reported a median EQ-5D HU score of 0.796, while our mean study population’s EQ-5D score was 0.78. The 2 study populations also had similar minimum and maximum EQ-5D score ranges, which represent substantial variation—the minimum EQ-5D score reported in both studies was −0.59 (representing health states worse than death) while the maximum EQ-5D score reported in both studies was 1.0 (representing optimal health) [19].

A major strength of our study was that we examined 2 large cohorts of heterogeneous PMO women who represent non-EU and EU countries, and we were able to compare 2 parallel sets of instruments that measure HRQoL and HU. This research has been precedent-setting. Another strength of this study was that we were able to measure the impact of important patient baseline characteristics, such as prevalent fractures, on the variability of HU and HRQoL scores. Our research is consistent with prior research on the effects of prevalent and incident vertebral and non-vertebral fractures on HRQoL and HU scores in separate and distinct populations [7,8,10-12].

There are important limitations to our study that warrant further discussion. First, we examined baseline data only; therefore, we did not compare correlations over time nor did we attempt to explain changes in health utility scores over time. Second, the results were based on a sample of patients from specific countries within the EU and from English-speaking countries outside the EU. The HUI results from the non-EU cohort, for instance, were substantially weighted by responses from the US patients given its large proportion of this cohort. Therefore, caution should be exercised when attempting to generalize these results to other patient populations.

Despite these limitations, the results of this study provide an important contribution toward characterizing the associations between disease-targeted, generic HRQoL/health status instruments and preference-based HU measures in PMO women. In addition, the study demonstrates that the number of vertebral and non-vertebral fractures is well correlated with HRQoL and HU scores. The OPAQ, QualEFFO, EQ-5D, HUI and, to a lesser degree, NHP scores increased or decreased significantly and in direct and linear proportion to the number of vertebral and non-vertebral fractures.

## Conclusions

Self-reported HRQoL and HU scores among postmenopausal women were substantially impacted by the number of prevalent vertebral fractures and, to a lesser but still significant degree, non-vertebral fractures. Our study found relatively strong correlations between multiple disease-specific HRQoL and HU instruments. A better understanding of how disease-specific HRQoL and HU measures are related may help to guide and inform proper instrument selection for randomized clinical trials and to provide data to develop more sensitive patient-reported outcomes instruments for the osteoporosis population and key subgroups of interest.

## Competing interests

Russel Burge, Wei Shen, April N. Naegeli, and Jahangir Alam are full-time employees of Eli Lilly and Company. Jeremy Shih is employed by inVentiv Clinical Solutions, which is a subcontractor to Eli Lilly and Company. Stuart Silverman and Deborah Gold have no competing interests.

## Authors’ contributions

RB and AN participated in the study design and helped draft the manuscript. WS participated in the study design and statistical analysis, and drafting of the manuscript. JA and JS conducted statistical analyses and review of the manuscript. SS and DG participated in the study design and provided important intellectual content for the manuscript. All authors read and approved the final manuscript.

## Supplementary Material

Additional file 1 **a – Comparison of utility scores by vertebral fracture subgroups – Non-EU cohort.** File provided as a Microsoft Word document with a .doc extension. **b –** Comparison of utility scores by vertebral fracture subgroups – EU cohort. File provided as a Microsoft Word document with a .doc extension. Click here for file

Additional file 2 **a – Comparison of utility domains and scores by non-vertebral fracture subgroups – Non-EU cohort.** File provided as a Microsoft Word document with a .doc extension. **b – **Comparison of utility domains and scores by non-vertebral fracture subgroups – EU cohort. File provided as a Microsoft Word document with a .doc extension. Click here for file
