# Supplementary material for: Use of health-related quality of life measures to predict health utility in postmenopausal osteoporotic women: results from the Multiple Outcomes of Raloxifene Evaluation study
Source: Health Qual Life Outcomes. 2013 Nov 5;11:189. doi: 10.1186/1477-7525-11-189 (PMC4228260; doi:10.1186/1477-7525-11-189)
Supplement: Additional file 1 — a – Comparison of utility scores by vertebral fracture subgroups – Non-EU cohort. File provided as a Microsoft Word document with a .doc extension. b – Comparison of utility scores by vertebral fracture subgroups – EU cohort. File provided as a Microsoft Word document with a .doc extension. [file 1477-7525-11-189-S1.docx]

**Additional file 1a – Comparison of utility scores by vertebral fracture subgroups – Non-EU cohort**

|  | **0 Vertebral Fractures** | | **1 Vertebral Fractures** | | **2 Vertebral Fractures** | | **3+ Vertebral Fractures** | | **p-values** | |
| --- | --- | --- | --- | --- | --- | --- | --- | --- | --- | --- |
| **Domain** | **N** | **Mean (SD)** | **N** | **Mean (SD)** | **N** | **Mean (SD)** | **N** | **Mean (SD)** | **Linear Trend** | **Overall** |
| **OPAQ Domains** |  |  |  |  |  |  |  |  |  |  |
| Walking/Bending | 260 | 87.4 (16.2) | 250 | 86.4 (16.2) | 104 | 84.8 (15.9) | 79 | 76.8 (21.3) | <0.001 | <0.001 |
| Standing/Sitting | 261 | 85.5 (18.0) | 248 | 79.4 (20.7) | 104 | 77.7 (20.5) | 79 | 68.1 (24.7) | <0.001 | <0.001 |
| Dressing/Reaching | 260 | 94.1 (14.9) | 248 | 93.6 (13.4) | 104 | 93.3 (14.4) | 79 | 87.9 (17.9) | 0.001 | 0.009 |
| Household/Self Care | 261 | 95.4 (10.8) | 248 | 93.1 (12.4) | 104 | 91.0 (14.5) | 79 | 88.9 (14.6) | <0.001 | <0.001 |
| Transfers | 261 | 93.6 (14.6) | 249 | 89.7 (19.4) | 104 | 90.0 (17.1) | 79 | 84.7 (24.6) | <0.001 | 0.001 |
| Usual Work | 261 | 94.1 (13.8) | 248 | 92.5 (14.2) | 104 | 90.6 (19.8) | 79 | 85.4 (19.5) | <0.001 | <0.001 |
| Fear of Falls | 260 | 76.9 (18.3) | 249 | 72.9 (19.3) | 104 | 72.3 (18.6) | 79 | 65.3 (22.2) | <0.001 | <0.001 |
| Level of Tension | 259 | 65.4 (16.7) | 248 | 70.5 (17.1) | 104 | 68.6 (17.4) | 79 | 67.5 (19.3) | 0.521 | 0.010 |
| Body Image | 261 | 70.6 (23.6) | 249 | 67.2 (24.8) | 104 | 66.3 (23.8) | 79 | 57.7 (27.4) | <0.001 | <0.001 |
| Independence | 260 | 86.6 (14.7) | 249 | 83.6 (17.6) | 104 | 82.8 (15.9) | 78 | 76.8 (22.4) | <0.001 | <0.001 |
| Back Pain | 261 | 79.9 (21.0) | 250 | 72.0 (24.2) | 104 | 71.4 (22.9) | 79 | 61.7 (25.7) | <0.001 | <0.001 |
| Fatigue | 261 | 63.5 (19.3) | 249 | 62.1 (18.1) | 104 | 64.2 (20.2) | 79 | 58.5 (16.6) | 0.094 | 0.161 |
| Social Activity | 260 | 39.5 (19.7) | 249 | 42.4 (19.0) | 104 | 37.1 (19.7) | 79 | 41.9 (18.9) | 0.783 | 0.086 |
| Support, Family and Friends | 260 | 84.2 (20.5) | 249 | 85.8 (20.6) | 103 | 85.0 (20.7) | 79 | 89.4 (17.5) | 0.071 | 0.244 |
| **NHP Domains** |  |  |  |  |  |  |  |  |  |  |
| Emotional Reaction | 256 | 5.6 (12.8) | 246 | 4.3 (11.7) | 102 | 4.1 (10.4) | 77 | 7.2 (16.4) | 0.378 | 0.242 |
| Energy | 261 | 10.7 (22.8) | 247 | 11.2 (22.6) | 102 | 9.7 (21.7) | 78 | 17.8 (28.3) | 0.036 | 0.081 |
| Physical Mobility | 259 | 6.8 (11.4) | 247 | 9.5 (13.2) | 102 | 10.4 (13.3) | 78 | 17.7 (16.8) | <0.001 | <0.001 |
| Pain | 257 | 9.1 (17.4) | 243 | 13.0 (22.7) | 100 | 11.1 (20.1) | 78 | 18.8 (28.1) | 0.001 | 0.004 |
| Sleep | 261 | 19.4 (24.7) | 247 | 19.0 (24.7) | 101 | 19.0 (24.6) | 78 | 21.6 (28.0) | 0.521 | 0.876 |
| Social Interaction | 260 | 2.6 (10.2) | 245 | 3.2 (10.8) | 101 | 1.7 ( 5.7) | 78 | 3.3 ( 9.9) | 0.852 | 0.566 |
| **MHUI Utility Score** | 261 | 0.9 ( 0.1) | 250 | 0.9 ( 0.1) | 104 | 0.8 ( 0.1) | 79 | 0.8 ( 0.1) | 0.006 | 0.050 |

Abbreviations: HUI = McMaster Health Utility Index; NHP = Nottingham Health Profile OPAQ = Osteoporosis Patient Assessment Questionnaire; SD = standard deviation.

Note: *p* values from analysis of variance (ANOVA).

**Additional file 1b – Comparison of utility scores by vertebral fracture subgroups – EU cohort**

|  | **0 Vertebral Fractures** | | **1 Vertebral Fractures** | | **2 Vertebral Fractures** | | **3+ Vertebral Fractures** | | **p-values** | |
| --- | --- | --- | --- | --- | --- | --- | --- | --- | --- | --- |
| **Domain** | **N** | **Mean (SD)** | **N** | **Mean (SD)** | **N** | **Mean (SD)** | **N** | **Mean (SD)** | **Linear Trend** | **Overall** |
| **EQ-5D Utility Score #** | 272 | 0.8 (0.2) | 124 | 0.7 (0.2) | 62 | 0.7 (0.3) | 93 | 0.7 (0.3) | <0.001 | <0.001 |
| **QualEFFO Domains** |  |  |  |  |  |  |  |  |  |  |
| Pain | 265 | 76.2 (24.3) | 122 | 65.6 (27.0) | 62 | 68.3 (25.8) | 92 | 51.8 (28.2) | <0.001 | <0.001 |
| Daily Activity | 272 | 94.0 (10.5) | 124 | 90.3 (13.5) | 62 | 91.7 (13.8) | 92 | 83.9 (19.3) | <0.001 | <0.001 |
| Mobility | 272 | 82.2 (17.5) | 124 | 76.6 (19.6) | 62 | 78.2 (18.3) | 92 | 66.8 (22.1) | <0.001 | <0.001 |
| General Health | 271 | 63.1 (20.2) | 124 | 55.9 (19.8) | 62 | 54.2 (20.2) | 91 | 49.9 (21.7) | <0.001 | <0.001 |
| Mental Health | 272 | 77.0 (16.1) | 124 | 75.4 (15.3) | 62 | 68.6 (18.9) | 91 | 72.5 (18.3) | 0.002 | 0.002 |
| Overall QOL | 259 | 77.5 (18.6) | 118 | 74.1 (19.0) | 60 | 66.5 (22.4) | 87 | 65.2 (20.9) | <0.001 | <0.001 |
| **NHP Domains** |  |  |  |  |  |  |  |  |  |  |
| Emotional Reaction | 260 | 8.3 (16.5) | 119 | 9.7 (18.1) | 59 | 15.7 (23.6) | 87 | 12.1 (20.5) | 0.019 | 0.029 |
| Energy | 269 | 13.6 (27.2) | 124 | 14.2 (27.5) | 62 | 23.5 (35.9) | 92 | 24.2 (32.7) | <0.001 | 0.004 |
| Physical Mobility | 262 | 10.3 (15.7) | 121 | 13.8 (16.7) | 61 | 14.6 (19.1) | 89 | 24.8 (23.3) | <0.001 | <0.001 |
| Pain | 263 | 15.7 (24.6) | 116 | 18.2 (24.6) | 60 | 21.9 (28.2) | 87 | 27.7 (30.7) | <0.001 | 0.002 |
| Sleep | 267 | 22.1 (27.4) | 120 | 25.5 (30.0) | 60 | 26.2 (29.2) | 89 | 28.4 (29.8) | 0.090 | 0.287 |
| Social Interaction | 266 | 3.9 (13.1) | 121 | 5.2 (13.5) | 60 | 10.9 (19.9) | 86 | 6.4 (16.9) | 0.027 | 0.010 |

Abbreviations: EQ=5D = EuroQol Group-Five Dimensions of HRQoL; NHP = Nottingham Health Profile; QualEFFO = European Foundation of Osteoporosis Quality-of-Life Assessment; SD = standard deviation.

Note: p-values from analysis of variance (ANOVA).
