# Supplementary material for: Use of health-related quality of life measures to predict health utility in postmenopausal osteoporotic women: results from the Multiple Outcomes of Raloxifene Evaluation study
Source: Health Qual Life Outcomes. 2013 Nov 5;11:189. doi: 10.1186/1477-7525-11-189 (PMC4228260; doi:10.1186/1477-7525-11-189)
Supplement: Additional file 2 — a – Comparison of utility domains and scores by non-vertebral fracture subgroups – Non-EU cohort. File provided as a Microsoft Word document with a .doc extension. b – Comparison of utility domains and scores by non-vertebral fracture subgroups – EU cohort. File provided as a Microsoft Word document with a .doc extension. [file 1477-7525-11-189-S2.docx]

**Additional file 2a – Comparison of utility domains and scores by non-vertebral fracture subgroups – Non-EU cohort**

|  | **0 Non- Vertebral Fractures** | | **1 Non- Vertebral Fractures** | | **2 Non- Vertebral Fractures** | | **3+ Non- Vertebral Fractures** | | **p-values** | |
| --- | --- | --- | --- | --- | --- | --- | --- | --- | --- | --- |
| **Domain** | **N** | **Mean (SD)** | **N** | **Mean (SD)** | **N** | **Mean (SD)** | **N** | **Mean (SD)** | **Linear Trend** | **Overall** |
| **OPAQ Domains** |  |  |  |  |  |  |  |  |  |  |
| Walking/Bending | 327 | 87.1 (15.4) | 193 | 85.3 (17.6) | 100 | 84.1 (18.0) | 73 | 79.9 (20.3) | <0.001 | 0.009 |
| Standing/Sitting | 327 | 82.1 (19.8) | 192 | 79.8 (20.4) | 100 | 78.9 (21.1) | 73 | 74.0 (24.8) | 0.003 | 0.020 |
| Dressing/Reaching | 327 | 93.9 (14.5) | 192 | 91.8 (16.2) | 100 | 93.8 (13.4) | 72 | 92.4 (13.4) | 0.680 | 0.418 |
| Household/Self Care | 327 | 95.3 ( 9.8) | 192 | 91.2 (14.3) | 100 | 92.1 (13.9) | 73 | 90.0 (15.4) | 0.003 | <0.001 |
| Transfers | 328 | 93.1 (14.5) | 192 | 90.3 (18.7) | 100 | 89.5 (18.7) | 73 | 82.4 (27.6) | <0.001 | <0.001 |
| Usual Work | 326 | 93.6 (14.0) | 193 | 90.7 (17.2) | 100 | 91.8 (14.7) | 73 | 88.7 (20.4) | 0.033 | 0.046 |
| Fear of Falls | 328 | 76.8 (17.8) | 191 | 73.6 (19.2) | 100 | 70.8 (20.1) | 73 | 61.6 (21.3) | <0.001 | <0.001 |
| Level of Tension | 327 | 68.2 (17.1) | 191 | 67.4 (17.6) | 99 | 70.1 (16.7) | 73 | 65.2 (18.9) | 0.358 | 0.310 |
| Body Image | 327 | 69.4 (25.2) | 193 | 67.0 (23.5) | 100 | 65.8 (25.1) | 73 | 60.3 (24.8) | 0.004 | 0.033 |
| Independence | 326 | 85.5 (15.4) | 193 | 82.0 (20.0) | 100 | 84.1 (15.7) | 72 | 80.9 (18.2) | 0.099 | 0.068 |
| Back Pain | 328 | 76.3 (23.2) | 193 | 73.6 (22.3) | 100 | 69.8 (23.9) | 73 | 67.6 (27.6) | 0.002 | 0.009 |
| Fatigue | 327 | 64.5 (18.5) | 193 | 60.9 (19.3) | 100 | 60.3 (18.9) | 73 | 60.8 (17.7) | 0.118 | 0.066 |
| Social Activity | 328 | 40.2 (18.6) | 191 | 39.6 (20.3) | 100 | 42.3 (20.9) | 73 | 41.1 (19.0) | 0.510 | 0.718 |
| Support, Family and Friends | 327 | 84.5 (20.5) | 192 | 86.1 (20.2) | 99 | 87.5 (18.6) | 73 | 85.4 (21.6) | 0.616 | 0.590 |
| **NHP Domains** |  |  |  |  |  |  |  |  |  |  |
| Emotional Reaction | 322 | 4.4 (12.4) | 188 | 5.9 (13.4) | 98 | 3.8 (10.1) | 73 | 7.8 (13.9) | 0.115 | 0.098 |
| Energy | 325 | 10.1 (22.4) | 190 | 12.5 (23.8) | 100 | 11.9 (21.0) | 73 | 15.2 (28.5) | 0.121 | 0.325 |
| Physical Mobility | 326 | 7.9 (12.2) | 189 | 9.2 (13.8) | 99 | 11.4 (13.4) | 72 | 15.3 (15.8) | <0.001 | <0.001 |
| Pain | 318 | 9.9 (19.6) | 189 | 12.6 (21.3) | 99 | 13.4 (22.7) | 72 | 16.8 (26.2) | 0.013 | 0.059 |
| Sleep | 325 | 18.9 (24.9) | 190 | 19.6 (25.9) | 100 | 19.1 (22.3) | 72 | 22.1 (27.6) | 0.386 | 0.813 |
| Social Interaction | 324 | 2.5 (10.3) | 189 | 2.4 ( 8.5) | 100 | 3.1 (10.1) | 71 | 4.1 (11.1) | 0.161 | 0.571 |
| **MHUI Utility Score** | 328 | 0.9 ( 0.1) | 193 | 0.8 ( 0.1) | 100 | 0.8 ( 0.1) | 73 | 0.8 ( 0.1) | 0.004 | 0.018 |

Abbreviations: HUI = McMaster Health Utility Index; NHP = Nottingham Health Profile; OPAQ = Osteoporosis Patient Assessment Questionnaire; SD = standard deviation.

Note: p-values from analysis of variance (ANOVA).

**Additional file 2b – Comparison of utility domains and scores by non-vertebral fracture subgroups – EU cohort**

|  | **0 Non- Vertebral Fractures** | | **1 Non- Vertebral Fractures** | | **2 Non- Vertebral Fractures** | | **3+ Non- Vertebral Fractures** | | **p-values** | |
| --- | --- | --- | --- | --- | --- | --- | --- | --- | --- | --- |
| **Domain** | **N** | **Mean (SD)** | **N** | **Mean (SD)** | **N** | **Mean (SD)** | **N** | **Mean (SD)** | **Linear Trend** | **Overall** |
| **EQ-5D Utility Score #** | 349 | 0.8 ( 0.2) | 122 | 0.8 ( 0.2) | 47 | 0.8 ( 0.2) | 33 | 0.7 ( 0.3) | 0.164 | 0.322 |
| **QualEFFO Domains** |  |  |  |  |  |  |  |  |  |  |
| Pain | 341 | 70.2 (27.2) | 121 | 65.4 (25.6) | 47 | 70.7 (28.3) | 32 | 63.4 (31.2) | 0.336 | 0.226 |
| Daily Activity | 349 | 91.7 (13.4) | 121 | 90.7 (12.5) | 47 | 92.5 (11.2) | 33 | 85.3 (22.6) | 0.025 | 0.067 |
| Mobility | 349 | 79.3 (19.2) | 121 | 74.7 (20.3) | 47 | 80.3 (16.4) | 33 | 71.1 (23.4) | 0.089 | 0.020 |
| General Health | 348 | 59.9 (21.1) | 121 | 54.2 (20.3) | 47 | 60.5 (20.0) | 32 | 51.8 (20.8) | 0.133 | 0.015 |
| Mental Health | 349 | 75.4 (16.1) | 121 | 73.4 (17.2) | 47 | 78.7 (18.2) | 32 | 70.9 (20.4) | 0.413 | 0.146 |
| Overall QOL | 331 | 74.4 (19.9) | 116 | 72.3 (18.8) | 46 | 73.4 (22.2) | 31 | 67.4 (23.5) | 0.095 | 0.269 |
| **NHP Domains** |  |  |  |  |  |  |  |  |  |  |
| Emotional Reaction | 334 | 9.4 (18.4) | 114 | 10.8 (17.1) | 46 | 11.1 (20.3) | 31 | 13.1 (23.0) | 0.307 | 0.671 |
| Energy | 347 | 17.0 (31.1) | 121 | 13.9 (23.7) | 47 | 18.6 (33.8) | 32 | 19.7 (27.4) | 0.462 | 0.655 |
| Physical Mobility | 337 | 13.1 (18.1) | 118 | 16.0 (19.2) | 46 | 13.0 (16.0) | 32 | 17.4 (22.2) | 0.361 | 0.344 |
| Pain | 332 | 18.0 (26.4) | 117 | 20.4 (24.8) | 46 | 18.7 (25.3) | 31 | 23.8 (33.9) | 0.314 | 0.599 |
| Sleep | 339 | 23.5 (28.7) | 118 | 24.6 (28.0) | 47 | 28.6 (30.9) | 32 | 26.6 (27.3) | 0.421 | 0.673 |
| Social Interaction | 337 | 5.1 (15.1) | 119 | 5.7 (14.2) | 46 | 5.8 (16.1) | 31 | 6.9 (14.0) | 0.520 | 0.899 |

Abbreviations: EQ-5D = EuroQol Group-Five Dimensions of HRQoL; NHP = Nottingham Health Profile; QualEFFO = European Foundation of Osteoporosis Quality-of-Life Assessment; SD = standard deviation.

Note: p-values from analysis of variance (ANOVA).
